# Retrospective analysis of clinical characteristics and outcomes of patients with carcinoma of unknown primary from three tertiary centers in Australia

**DOI:** 10.1002/cam4.7052

**Published:** 2024-03-25

**Authors:** Emma L. Boys, Bo Gao, Peter Grimison, Sarah Sutherland, Karen L. MacKenzie, Roger R. Reddel, Jia Liu

**Affiliations:** ^1^ ProCan®, Children's Medical Research Institute Westmead New South Wales Australia; ^2^ Faculty of Medicine and Health The University of Sydney Sydney New South Wales Australia; ^3^ Department of Medical Oncology Crown Princess Mary Cancer Centre Westmead New South Wales Australia; ^4^ Blacktown Cancer and Haematology Centre, Blacktown Hospital Blacktown New South Wales Australia; ^5^ Chris O'Brien Lifehouse Sydney New South Wales Australia; ^6^ School of Medical Science, Faculty of Medicine and Health The University of Sydney Sydney New South Wales Australia; ^7^ Sydney Medical School, Faculty of Medicine and Health The University of Sydney Sydney New South Wales Australia; ^8^ The Kinghorn Cancer Centre, St Vincent's Hospital Darlinghurst New South Wales Australia; ^9^ School of Clinical Medicine, St Vincent's Campus University of New South Wales Sydney New South Wales Australia

**Keywords:** cancer of unknown primary site, empirical therapy, real‐world data, site‐specific therapy

## Abstract

**Background:**

Carcinoma of unknown primary (CUP) remains an important tumor entity and a disproportionate cause of cancer mortality. Little is known about the contemporary clinical characteristics, treatment patterns, and outcomes of CUP patients based on updated international classification guidelines. We evaluated a contemporary CUP cohort to provide insight into current clinical practice and the impact of tissue of origin assignment, site‐specific and empirical therapy in a real‐world setting.

**Methods:**

We conducted a retrospective cohort study of CUP patients, as defined by the updated European Society of Medical Oncology (ESMO) 2023 guidelines, across three tertiary referral centers in Australia between 2015 and 2022. We analyzed clinical characteristics, treatment patterns, and survival outcomes using the Kaplan–Meier method and Cox regression proportional hazard model between favorable and unfavorable risk groups.

**Results:**

We identified a total of 123 CUP patients (*n* = 86 unfavorable, *n* = 37 favorable risk as per the 2023 ESMO guidelines). Sixty‐four patients (52%) were assigned a tissue of origin by the treating clinician. Median progression free survival (PFS) was 6.8 (95% confidence interval (CI) 5.1–12.1) months and overall survival (OS) 10.2 (95% CI 6.0–18.5) months. Unfavorable risk (hazard ratio [HR] 2.9, *p* = 0.006), poor performance status (HR 2.8, *p* < 0.001), and non‐squamous histology (HR 2.5, *p* < 0.05) were associated with poor survival outcome. A total of 70 patients (57%) proceeded to systemic therapy. In patients with non‐squamous histology and unfavorable risk, site‐specific therapy compared to empirical chemotherapy did not improve outcome (median OS 8.2 vs. 11.8 months, *p* = 0.7).

**Conclusions:**

In this real‐world cohort, CUP presentations were heterogenous. Overall survival and rates of systemic treatment were poor. Poor performance status and unfavorable risk were associated with worse survival. For most patients, site‐specific therapy did not improve survival outcome. Improved and timely access to diagnostic tests and therapeutics for this group of patients is urgently required.

## 1 INTRODUCTION

Carcinoma of unknown primary (CUP) is a heterogenous group of histologically confirmed metastatic cancers where the primary site remains elusive despite appropriate clinical investigations.^1^ Diagnostic advances have resulted in declining CUP incidence. CUP previously accounted for 3%–5% of all malignancies, with rates falling to 1%–2% over the last several decades.^2^, ^3^ Diagnostic improvements, however have not yet manifested in a survival advantage. CUP accounts for a disproportionate degree of mortality and remains within the top five causes of cancer death with little improvement in survival rates over time.^1^, ^4^, ^5^, ^6^, ^7^

The recently updated European Society of Medical Oncology (ESMO) guidelines stratify CUP patients into favorable and unfavorable subsets. Favorable subsets include well recognized clinical entities consistent with a site of origin, and for which effective treatments are available such as colorectal, breast, and recently, renal‐like CUP.^1^ These patients have survival akin to their counterparts with known primary sites.^5^, ^6^, ^8^ Unfavorable CUP is more common, accounting for approximately 80% of all CUP cases and is characterized by visceral metastatic disease and limited treatment response. Empirical platinum doublet chemotherapy is recommended for these patients with poor median overall survival (OS) of less than 12 months.^6^, ^8^, ^9^, ^10^, ^11^, ^12^, ^13^

The recommended clinical workup of CUP patients involves comprehensive history and examination, basic blood tests and computed tomography (CT) imaging of the chest, abdomen, and pelvis. Women should also have mammography. Other investigations such as whole body 2‐deoxy‐2‐[18F] fluoro‐D‐glucose‐positron emission tomography (FDG‐PET), tests for tumor markers and upper and lower gastrointestinal endoscopy are considered depending on the clinical context. The cornerstone of diagnosis remains tissue biopsy for histopathology and immunohistochemistry (IHC) assessment to attempt to delineate the broad type of malignancy and tissue of origin.^1^

Genomic testing to identify both tissue of origin and actionable treatment alterations, as recommended by the National Comprehensive Cancer Network and the United Kingdom National Health Service, has been actively explored and is anticipated to play an increasing role in the workup of CUP patients.^14^, ^15^ However, to date, the use of site‐ or molecular‐directed treatment has not been demonstrated to improve OS in CUP patients and therefore these tests are not universally recommended.^1^ Further, access to and reimbursement for genomic testing is not uniformly available and in certain jurisdictions, access depends on participation in clinical trials or self‐funding at great expense.^16^

Little is known about the contemporary patient characteristics and patterns of care of CUP patients, as redefined by the recently updated ESMO guidelines. In this retrospective cohort study, we aimed to evaluate the clinical characteristics, treatment patterns and outcomes of patients with CUP to inform clinical practice. We specifically aimed to evaluate the role of tissue of origin assignment and site‐directed treatment in a real‐world setting.

## 2 METHODS

### 2.1 Study design and patients

Patients with CUP, as defined by the 2023 ESMO guidelines,^1^ who received treatment at Chris O'Brien Lifehouse, Westmead, or Blacktown Hospitals in Sydney, Australia between January 2015 and December 2022 were included (Table S1). Patients were identified via a medical record database search for the International Classification of Diseases 10th Revision (ICD‐10) codes (C76, C78, C79, and C80) and combinations of the keywords “cancer”, “carcinoma”, and “unknown primary”. In accordance with the ESMO guidelines, patients were classified into favorable and unfavorable risk subsets. Patients were excluded if they did not receive a standardized workup consisting of history, physical examination, basic blood tests, CT imaging of the chest, abdomen and pelvis, and tissue biopsy. The absence of mammography was not an exclusion criterion. As per the ESMO guidelines,^1^ patients were also excluded if histological subtype was documented as sarcoma, melanoma, germ cell, neuroendocrine, or lymphoma.

From the medical record, we collected data on baseline demographics, Eastern Cooperative Oncology Group (ECOG) performance status, molecular testing, histopathology (including histological type, IHC stains performed, and time taken for biopsy results) and treatment details (systemic therapy including targeted therapy or immunotherapy, radiotherapy and reasons for treatment ineligibility). Actionable molecular alterations were defined as per the tier 1 OncoKB classification.^17^ We also collected data on tissue of origin assignment by the treating clinician as documented in the medical record. This was determined by clinician judgment, molecular testing or receipt of a first line site specific systemic therapy. Best radiologic response to first line treatment was evaluated as per clinician assessment.

Approval was provided by the local Human Research Ethics Committee (2021/ETH01324) and a waiver of consent was granted given the low‐risk and retrospective nature of this study.

### 2.2 Statistical analysis

Baseline characteristics between favorable and unfavorable risk patients were compared using summary statistics (percentages and median). The Chi‐square test was used to compare tissue of origin assignment between favorable and unfavorable risk groups. Progression free survival (PFS) was defined as the time from diagnosis to clinical or radiological progression, or death. OS was defined as the time from diagnosis to death of any cause up to February 8, 2023. The follow‐up period for each patient commenced on the date of diagnosis until the occurrence of disease progression or death. For patients where disease progression or death was not observed, the follow‐up time was censored at the date of the last clinical assessment. The Kaplan–Meier method was used to estimate PFS and OS. The log rank test was used to compare groups. Univariable and multivariable analyses of variables associated with PFS and OS were performed using the Cox regression proportional hazard model. The proportional hazards assumption was assessed through visual inspection of Kaplan Meier curves and Schoenfeld residual plots. Variables with a *p* value <0.05 were included in the multivariable model. For all analyses, a *p* value of <0.05 was considered statistically significant. Statistical analysis was performed using R version 4.2.3 for Windows. The cutoff date for all data used in the analyses was February 8, 2023.

## 3 RESULTS

### 3.1 Baseline patient characteristics

**FIGURE 1:**
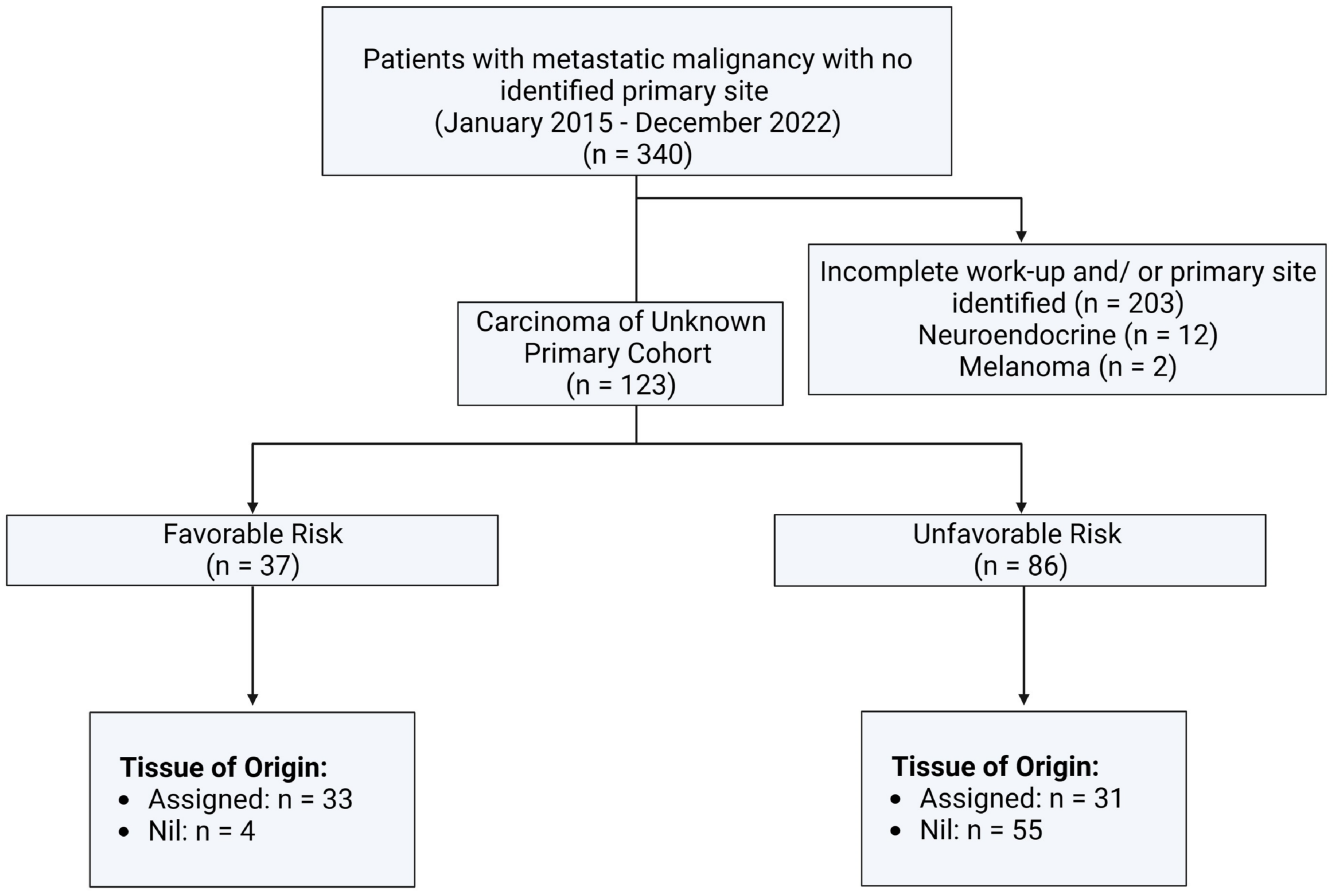
Study flowchart. Patients were classified into favorable and unfavorable risk groups as per the 2023 European Society of Medical Oncology (ESMO) guidelines (1).

Of 340 patients diagnosed with metastatic malignancy with no identified primary site in hospital databases between 2015 and 2022, 123 patients met the study inclusion criteria (Figure 1). Patient demographics and clinical features are summarized in Table 1. Most patients (70%) were classified as unfavorable risk. Median age was 65 years, 47% were female and 76% had an ECOG performance status of 0 or 1. A median number of 12 IHC stains were performed for cases of non‐squamous histology. The median time from biopsy to histopathology result was 5 days.

**TABLE 1:** Baseline patient characteristics for the study cohort, stratified by favorable and unfavorable risk.

	Favorable (*n* = 37)	Unfavorable (*n* = 86)	Total (*n* = 123)	*p*
Age (median, range)	65 (44–98)	64 (34–87)	65 (34–98)	
Gender (%)				
Male	20 (54)	44 (51)	64 (52)	
Female	17 (45)	42 (49)	59 (47)	
ECOG (%)				
0	24 (65)	24 (28)	48 (39)	
1	12 (32)	33 (38)	45 (37)	
2	0 (0)	15 (17)	15 (12)	
3	1 (3)	13 (15)	14 (11)	
Not recorded	0 (0)	1 (1)	1 (1)	
Previous cancer diagnosis (%)	6 (16)	10 (12)	16 (13)	
Smoking status (%)				
Current or former smoker	15 (41)	44 (51)	59 (48)	
Non‐smoker	14 (38)	26 (30)	40 (33)	
Not recorded	8 (22)	16 (19)	24 (20)	
Histological type (%)				
Adenocarcinoma	10 (27)	33 (38)	43 (35)	
Carcinoma, NOS	4 (11)	44 (51)	48 (39)	
Squamous	23 (62)	5 (6)	28 (23)	
Unknown	0 (0)	4 (5)	4 (3)	
Number of IHC stains (median, range)				
Squamous	1 (0–2)	1 (0–1)	1 (0–2)	
Non‐squamous	9 (0–27)	12 (0–32)	12 (0–32)	
Time to biopsy result (median [days], range)	5 (2–20)	5 (1–29)	5 (1–29)	
Biopsy site				
Liver	2 (5)	32 (37)	34 (28)	
Lymph node	22 (59)	23 (27)	45 (37)	
Other	9 (24)	29 (34)	38 (31)	
Unknown	4 (11)	2 (2)	6 (5)	
Systemic therapy	23 (62)	47 (55)	70 (57)	
Anti‐cancer treatment (any line) (%)				
Chemotherapy	23 (62)	47 (55)	70 (57)	
Immunotherapy	3 (8)	15 (17)	18 (15)	
Targeted therapy	4 (11)	3 (3)	7 (6)	
Radiotherapy	22 (59)	21 (24)	43 (35)	
Best response to first line treatment^a^				
Complete response	14 (61)	2 (4)	16 (23)	
Partial response or stable disease	9 (39)	32 (68)	41 (59)	
Progressive disease	0 (0)	13 (28)	13 (19)	
Tissue of origin assigned by treating clinician (%)	33 (89)	31 (36)	64 (52)	<0.001
Assigned tissue of origin (%)				
Anus	1 (3)	0 (0)	1 (1)	
Appendix	1 (3)	0 (0)	1 (1)	
Breast	1 (3)	0 (0)	1 (1)	
Colorectal^b^	7 (19)	2 (2)	9 (7)	
Esophagus	0 (0)	1 (1)	1 (1)	
Gallbladder Gastrointestinal, NOS	0 (0) 1 (3)	2 (2) 0 (0)	2 (2) 1 (1)	
Head and neck	1 (3)	2 (2)	3 (2)	
Lung	0 (0)	7 (8)	7 (6)	
Nil	4 (11)	55 (64)	59 (48)	
Oropharynx	17 (46)	2 (2)	19 (15)	
Ovary	1 (3)	0 (0)	1 (1)	
Pancreas	0 (0)	9 (10)	9 (7)	
Renal	1 (3)	0 (0)	1 (1)	
Skin	2 (5)	2 (2)	4 (3)	
Stomach	0 (0)	4 (5)	4 (3)	
Molecular testing (%)	5 (14)	20 (23)	25 (20)	
Actionable molecular alteration^c^	4 (11)	7 (8)	11 (9)	
Targeted therapy based on molecular testing	3 (8)	3 (3)	6 (5)	
First line empirical chemotherapy regimen	1 (3)	32 (37)	33 (47)	
Carboplatin/gemcitabine		25 (29)	25 (76)	
Carboplatin/paclitaxel		3 (3)	3 (9)	
Cisplatin/ gemcitabine	1 (3)	4 (5)	5 (15)	
First line site specific regimen	22 (59)	15 (17)	37 (53)	
5‐fluorouracil +/− oxaliplatin +/− irinotecan	7 (19)	8 (9)	15 (41)	
Anti‐PD1 or PD‐L1 inhibitor		5 (6)	5 (14)	
Carboplatin/5‐fluorouracil	1 (3)		1 (2)	
Carboplatin/etoposide		1 (1)	1 (2)	
Carboplatin/pemetrexed		1 (1)	1 (2)	
Cisplatin/gemcitabine		1 (1)	1 (2)	
Docetaxel	1 (3)		1 (2)	
Docetaxel/5‐fluorouracil/cisplatin	1 (3)		1 (2)	
Doxorubicin/ cyclophosphamide	1 (3)		1 (2)	
Epirubicin/cisplatin/capecitabine		1 (1)	1 (2)	
Gemcitabine/nab‐paclitaxel		3 (3)	3 (8)	
Platinum +/− taxane	11 (30)		11 (30)	
Targeted therapy	2 (9)		2 (5)	

Abbreviations: ECOG, eastern cooperative oncology group; IHC, immunohistochemistry; NOS, not otherwise specified.

^a^ Best response to first line treatment defined as per clinician assessment.

^b^ Two unfavorable risk patients had an atypical histology pattern that is not CK7 negative, CK20 positive, CDX2 positive but were assigned a colorectal site of origin by the treating clinician.

^c^ Based on tier 1 OncoKB classification (17).

### 3.2 Tissue of origin assignment

Sixty‐four (52%) of patients were assigned a likely tissue of origin based on clinical, imaging, histopathology, and/or molecular testing. Three patients (2%) underwent gene expression profiling as part of a clinical trial, which did not provide additional diagnostic clarity. Within the favorable risk category, oropharynx was the most assigned site of origin (46%). Within the unfavorable risk category, there was no clear predominant site assigned, however most patients were assigned an upper gastrointestinal (gallbladder, esophageal, pancreas, or stomach) site of origin (52%) (Table 1). Favorable risk patients were more likely to be assigned a tissue of origin compared to unfavorable risk patients (89% vs. 36%, *p* < 0.001).

### 3.3 Systemic treatment

Seventy patients (57%) proceeded to receive systemic therapy. All 70 patients received chemotherapy. In addition, 18 patients (15%) received immunotherapy and seven patients (6%) received targeted therapy. The most common reasons for treatment ineligibility were poor performance status (*n* = 17; 32%) or rapid symptomatic decline prior to treatment initiation (*n* = 9; 17%) (Table 2).

**TABLE 2:** Reasons for treatment ineligibility.

Reason for systemic treatment ineligibility	*n* (%)
Poor performance status	17 (32%)
Oligometastatic site treated with resection and/or radiotherapy	17 (32%)
Rapid symptomatic decline	9 (17%)
Unknown	6 (11%)
Patient declined treatment	4 (8%)
Total	53

**FIGURE 2:**
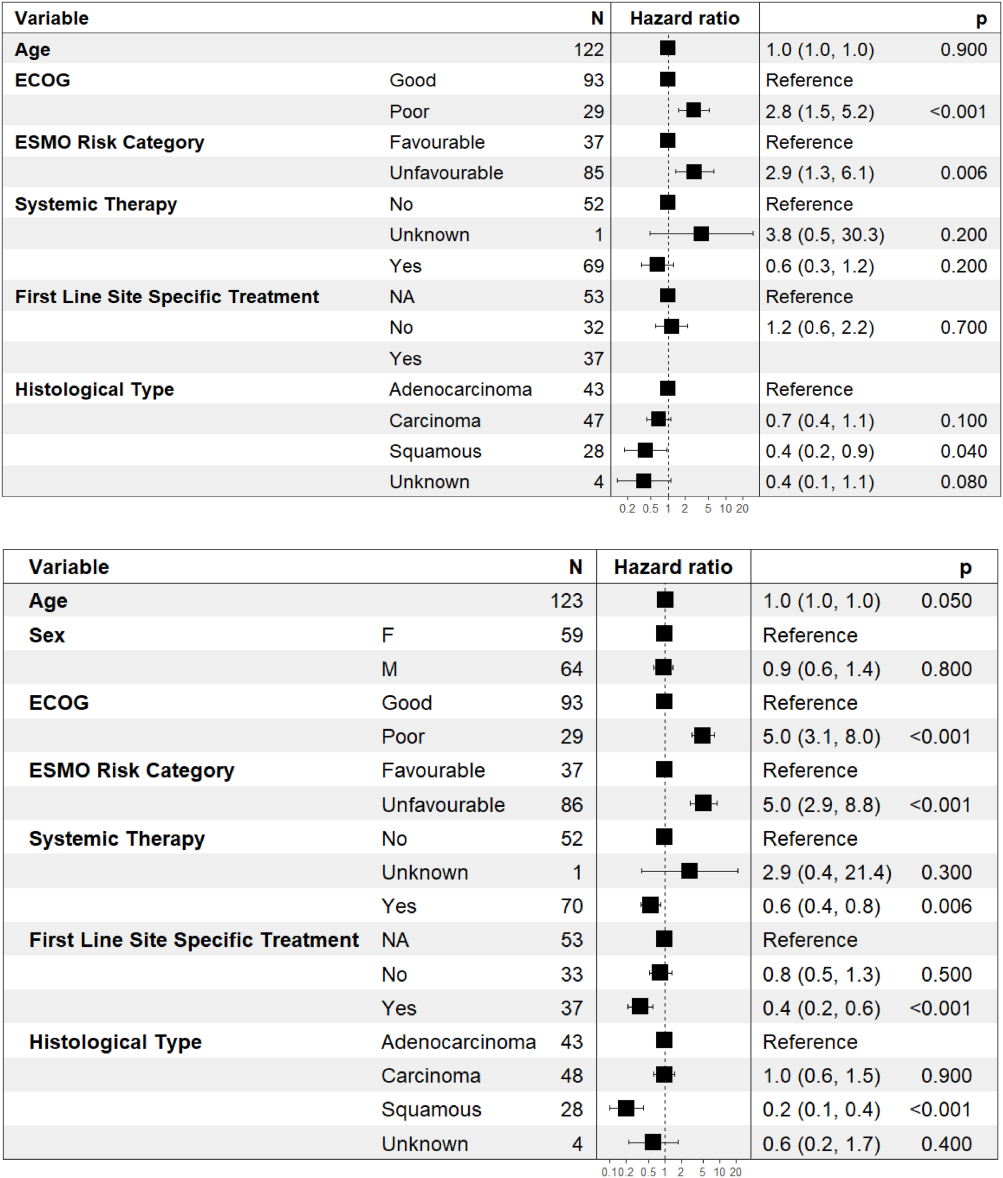
(A) Multivariable Cox regression proportional hazards analysis of clinical variables associated with overall survival (OS) in the entire study cohort. (B) Univariable Cox regression proportional hazards analysis of clinical variables associated with OS in the entire cohort. ECOG, Eastern Cooperative Oncology Group; ESMO, European Society of Medical Oncology.

Receipt of systemic treatment was associated with improved OS (median 18.5 (95% confidence interval (CI) 12.3–30.9) months vs. 4.3 (95% CI 2.3–9.3) months, *p =* 0.005) (Figure S1). However, when adjusted for age, performance status, histological type and site‐specific treatment, systemic therapy was not significantly associated with OS (HR 0.6 [95% CI 0.3–1.2], *p* = 0.2) (Figure 2A).

Patients who received targeted therapy or immunotherapy received a median of two lines of systemic treatment (range 1–6) compared to a median of one line of systemic treatment (range 1–4) in patients who received chemotherapy alone. OS was numerically greater with targeted or immunotherapy at 25.3 (95% CI 18.5–43.2) months compared to 14.3 (8.3–42.6) months in patients who received only chemotherapy, but this difference was not significant (*p* = 0.6) (Figure S2).

### 3.4 Molecular testing, immunotherapy, and targeted therapy

Twenty‐five patients (20%) underwent molecular testing. Of these patients, 11 (46%) had an actionable alteration. Six patients had therapy decisions made based on the presence of detected molecular alterations. An additional patient with a presumed renal cell carcinoma received treatment with sunitinib as second line therapy with OS of 10.5 months. Actionable molecular alterations, targeted therapies and relevant molecular targets are summarized in Table 3. Five patients received immunotherapy in the first line and 13 patients received immunotherapy in the second line or beyond. Three patients received treatment with combination anti‐PD1 and anti‐CTLA4 inhibitors, 13 patients received single agent anti‐PD1 or anti‐PDL1 therapy and two patients received immunotherapy as part of a clinical trial.

**TABLE 3:** Demographics, assigned tissue of origin, molecular target, and targeted agent for patients who had actionable molecular alterations and/or received targeted therapy.

Gender	Age	Risk category	Assigned tissue of origin	Molecular target identified	Targeted therapy	Line of therapy molecular treatment received
M	68	Favorable	Colorectal	RAS/RAF wild‐type	Cetuximab	3
M	66	Favorable	Colorectal	RAS/RAF wild‐type	Cetuximab	3
F	47	Favorable	Colorectal	KRAS G13D or G13N detected	Bevacizumab	1
M	66	Favorable	Renal	Nil	Sunitinib	2
M	39	Unfavorable	Gallbladder	FGF2R intron 17 rearrangement	Infigratinib	6
M	41	Unfavorable	Nil	BAP1 mutation	Olaparib	2
F	42	Unfavorable	Pancreas	BRAF V600E	Lifirafenib & dabrafenib/trametinib	3,4
F	34	Unfavorable	Nil	BRCA1 mutation	Nil	NA
M	57	Unfavorable	Gallbladder	IDH1 and BAP1 co‐mutation	Nil	NA
F	56	Unfavorable	Nil	FGFR2 fusion	Nil	NA
F	85	Favorable	Colorectal	KRAS G13D mutation	Nil	NA
M	61	Unfavorable	Colorectal	KRAS mutation	Nil	NA

### 3.5 Outcomes

In the overall cohort, median PFS was 6.8 (95% CI 5.1–12.1) months and OS 10.2 (95% CI 6.0–18.5) months (data not shown). In univariable analysis, poor performance status (HR 5.0, 95% CI 3.1–8.0, *p* < 0.001) was associated with worse survival. Squamous histology was associated with improved outcome (HR 0.2, 95% CI 0.1–0.4, *p* < 0.001) (Figure 2B). When adjusted for age, receipt of systemic therapy and site‐specific therapy, good performance status (ECOG 0 or 1) (HR 0.4, 95% CI 0.2–0.7, *p* < 0.001) and squamous histology (HR 0.4, 95% CI 0.2–0.9, *p* = 0.04) remained significantly associated with improved survival outcome (Figure 2A).

#### 3.5.1 Favorable versus unfavorable risk for non‐squamous histology

**FIGURE 3:**
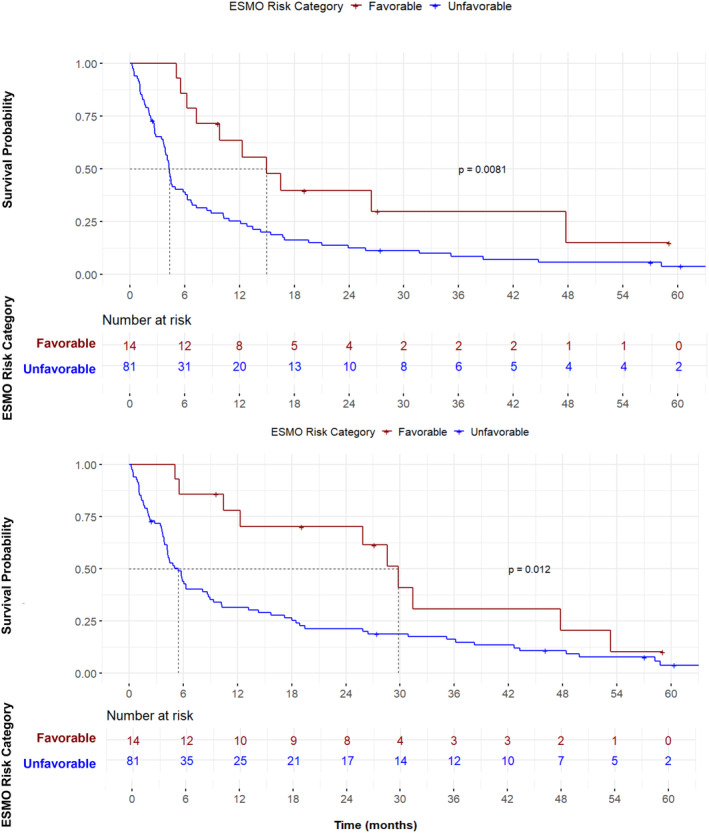
Survival of patients with non‐squamous histology stratified by ESMO risk category. (A). Progression free survival (PFS). Favorable risk patients (median 15.0, 95% CI 9.8—no estimate months) had improved PFS compared to unfavorable risk patients (median 4.3, 95% CI 3.9–6.1 months). (B). Overall survival (OS). Favorable risk patients (median 29.8, 95% CI 2.3—no estimate months) had improved overall survival compared to unfavorable risk patients (median 5.5, 95% CI 4.2–8.7 months).

Given that squamous histology was associated with improved survival outcome, we performed further analysis on patients with non‐squamous histology. In this group of patients, favorable risk was associated with improved survival outcome. Median PFS in favorable risk patients was 15.0 (95% CI 9.8–no estimate) months compared to 4.3 (95% CI 3.9–6.1) months in unfavorable risk patients (*p* = 0.008) (Figure 3A). Median OS in favorable risk patients was 29.8 (95% CI 12.3–no estimate) months compared to 5.5 (95% CI 4.2–8.7) months in unfavorable risk patients (*p*  = 0.01) (Figure 3B). The survival benefit conferred by favorable risk was maintained even when patients who did not receive systemic therapy were excluded from analysis (Figures S3 and S4).

Unfavorable risk (HR 2.2, 95% CI 1.0–4.6, *p* = 0.05) and poor performance status (HR 11.5, 95% CI 3.7–36.2, *p* < 0.001*)* were associated with poor survival in univariable analysis (HR 2.2, 95% CI 1.0–4.6, *p* = 0.05) (Figure S5).

#### 3.5.2 Site‐specific versus empirical chemotherapy for non‐squamous histology

A total of 31 (54%) and 26 (46%) patients with non‐squamous histology received empirical and site‐specific first line systemic therapy respectively. Prescribed therapy regimens are summarized in Table 1. The most common empirical chemotherapy regimen was carboplatin and gemcitabine (*n* = 25; 76%). Reflecting the predominance of gastrointestinal site of origin assignment, the most common site‐specific chemotherapy regimen was 5‐fluorouacil based (*n* = 15; 41%).

**FIGURE 4:**
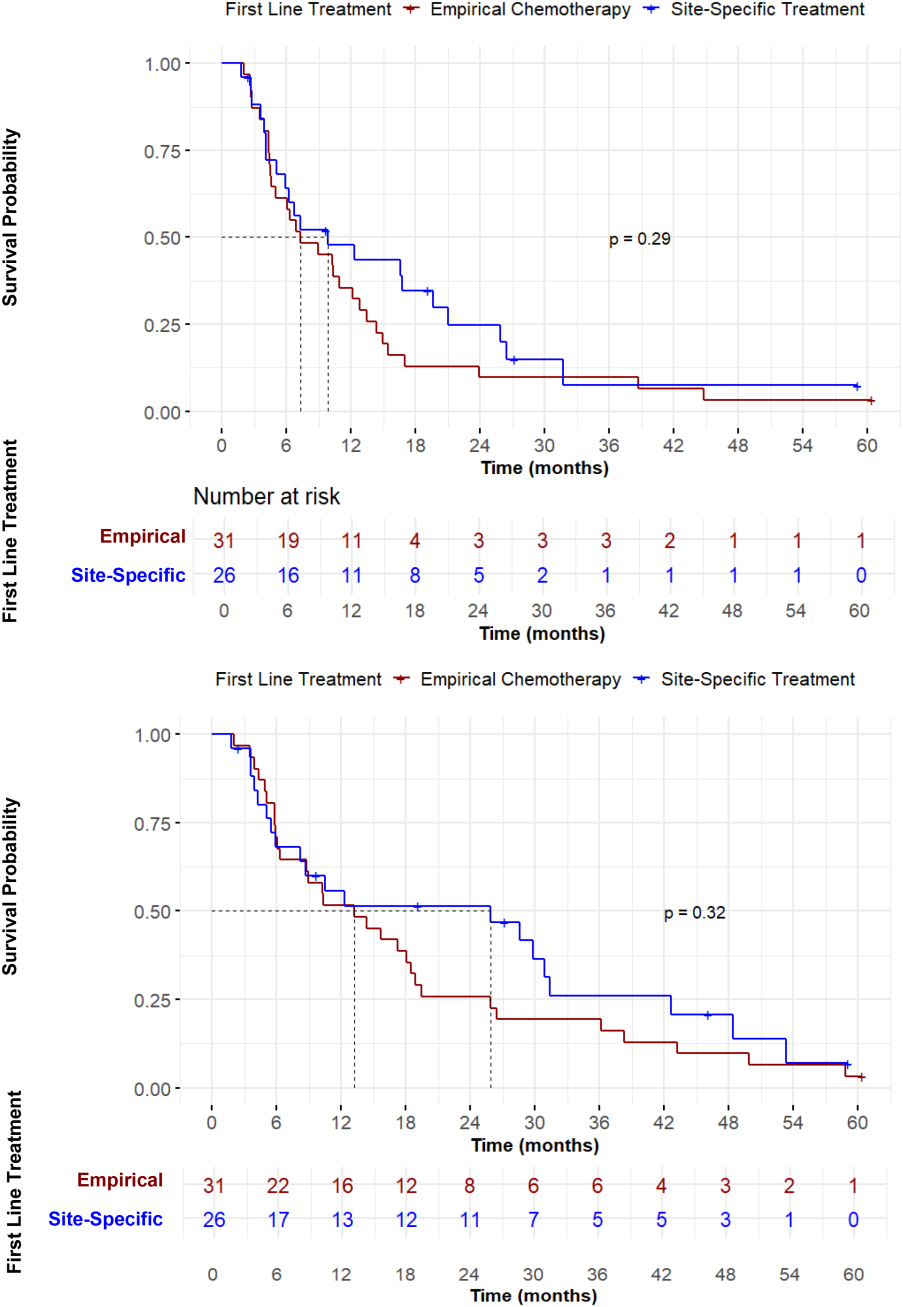
Survival of patients with non‐squamous histology stratified by site‐specific therapy. (A) PFS. Patients who received site‐specific therapy (median 9.8, 95% CI 5.9–25.8 months) had similar PFS compared to patients who received empirical therapy (median 7.3, 95% CI 4.6–13.5 months). (B) OS. Patients who received site‐specific therapy had numerically greater OS (median 25.9 months, 95% CI 8.2–42.6 months) compared to patients who received empirical chemotherapy (median 13.2, 95% CI 6.3–19.5 months) but this was not statistically significant (*p* = 0.3).

Receipt of site‐specific therapy did not improve survival outcome. Median PFS was 9.8 (95% CI 5.9–25.8) months with receipt of site‐specific therapy compared to 7.3 (95% CI 4.6–13.5) months with empirical therapy (*p* = 0.3) (Figure 4A). In patients who received site‐specific therapy, median OS was numerically greater at 25.9 (95% CI 8.2–42.6) months compared to 13.2 (95% CI 6.3–19.5) months in patients who received empirical therapy, but this was not statistically significant (*p* = 0.3) (Figure 4B). In the unfavorable risk group alone, patients who received site‐specific therapy had similar OS (8.2 months, 95% CI 4.2–no estimate) to patients who received empirical therapy (11.8 months, 95% CI 6.3–18.9) (*p* = 0.7) (Figure S6). In univariable analysis, first line site specific treatment was not associated with improved outcome (HR 0.7, 95%CI 0.4–1.3, *p* = 0.3) (Figure S5).

## 4 DISCUSSION

This study evaluated the characteristics, treatment patterns, and outcomes of patients with CUP seen at three Australian tertiary referral centers. There were several key findings. First, in a CUP cohort where most patients were of good performance status and underwent recommended diagnostic workup, only 57% of patients received systemic treatment. Second, we confirm that favorable risk patients have markedly improved survival outcomes compared to unfavorable risk patients, who account for most clinical presentations of CUP. Finally, site‐specific treatment, as compared to empirical chemotherapy did not improve survival outcome.

For patients with non‐squamous histology, irrespective of systemic treatment, survival outcomes were improved in the favorable risk group (median PFS 15.0 months vs. 4.3 months, *p* = 0.008; median OS 29.8 vs. 5.5 months, *p* = 0.01). Median OS in unfavorable risk patients who received systemic treatment was 10.2 months. This is comparable to recent published data from the only dedicated CUP clinic in Australia. In this study, 62% of patients underwent genomic profiling and 83% of patients received systemic therapy, with a median OS of 23.7 months in favorable risk patients compared to 10.9 months in unfavorable risk patients.^16^ Patients in our study also had similar survival outcomes compared to other contemporary cohorts which have reported median OS ranging from 1.2–4 months for patients who did not receive treatment and 9.5–12 months for patients who did receive treatment.^18^, ^19^, ^20^ Historical literature has reported a range of OS with palliative platinum‐based chemotherapy from 2.7 to 11 months in unfavorable risk patients.^9^, ^11^, ^12^, ^13^ Although this study only included patients who completed a standardized clinical workup, most of whom were of good performance status, survival outcomes remain poor. There remains an unmet need for more effective treatments for these patients.

Approximately half of our cohort (52%) were assigned a putative tissue of origin by the treating clinician. As only 20% of our cohort underwent molecular testing, this was largely based on clinical, imaging, or histopathology features alone. At the dedicated Australian CUP clinic, only 31% of cases were assigned a likely site of origin, however this was based on clinicopathologic criteria for over half of these cases.^16^ In a Japanese cohort, tissue of origin was assigned to 73% of patients based on an IHC panel alone.^19^ These differences may relate to the strength of evidence needed to support tissue of origin assignment within a dedicated and multidisciplinary CUP clinic, compared to routine oncology practice. This data also corroborates other studies in the Australian jurisdiction, where oncologists may assign a tissue of origin without definitive evidence of a primary site in part so that the patient can obtain access to pharmaceutical treatment.^21^, ^22^

Only 57% of patients within our cohort received systemic therapy. This is higher than reported literature rates where less than 50% of CUP patients proceed to systemic treatment.^18^, ^20^, ^23^ The higher rates of systemic therapy in this study in part reflect our specific study inclusion criteria which necessitated a complete diagnostic workup, including tissue biopsy, to confirm the diagnosis of CUP. Many previous studies, especially those that have relied on registry databases, have also included the population of frail patients with a clinical CUP diagnosis only, who are not fit for systemic treatment.^18^, ^23^ The most common reason for treatment ineligibility was poor performance status. However, a group of patients (*n* = 9; 17%) did not receive the intended treatment due to rapid symptomatic decline prior to treatment initiation. Similar challenges have been reported in previous CUP studies and highlight the importance of timely workup and assessment of these patients.^24^, ^25^

For patients with non‐squamous histology, site‐specific therapy did not improve outcomes. Randomized trial data conflict as to the impact of site‐specific versus empirical chemotherapy in CUP patients. As in our dataset, certain favorable subgroups benefit from this approach, but in the overall CUP population, evidence remains far from conclusive.^26^, ^27^, ^28^, ^29^ Two meta‐analyses from a series of heterogenous study populations and designs showed no survival improvement with site‐specific compared to empirical therapy.^26^, ^30^ However, in the more recent meta‐analysis, patients with responsive tumor types such as lung, kidney, and colorectal cancer benefited from this approach (HR 0.67, 95% CI 0.46–0.97). This effect was enhanced when accurate and well validated tissue of origin assays were used.^30^ Recent data from the Australian dedicated CUP clinic showed that site‐specific, targeted or immunotherapy improved survival outcome (median 20.2 vs. 10.9 months, *p* = 0.001).^16^ In our cohort, few patients received targeted therapy or immunotherapy, however these patients had numerically improved OS compared to patients treated with chemotherapy alone (median 25.3 vs. 14.3 months, *p* = 0.6). Most of these patients also received multiple lines of treatment. Such approaches warrant further exploration in CUP patients. Further, most patients in our study who received site‐specific treatment received treatment for an upper gastrointestinal site of origin. Like CUP, prognosis for this group of patients, even with systemic treatment is poor and may provide an explanation as to why site‐specific therapy did not result in improved outcomes. This is akin to the experience in the randomized GEFCAPI trial, where pancreaticobiliary tumors were the most assigned site, and no difference was found between site‐specific and empirical therapy.^24^ Similar findings were also noted within a retrospective Japanese cohort, where a large proportion of patients were assigned an upper gastrointestinal site of origin.^19^

The heterogenous nature of CUP as a disease entity across studied populations increases the difficulty of drawing conclusions regarding the role of site‐specific treatment. Although we did not find a survival difference with site‐specific treatment, we anticipate that over time more precise molecular subgroup definitions will be developed, and additional favorable subgroups will emerge. The recently updated ESMO guidelines, which includes addition of renal‐like CUP as a subset with favorable prognosis due to the large survival improvements seen with tyrosine kinase and immune checkpoint inhibitors, are an example of this.^1^ This is likely to result in an increasing subpopulation of CUP patients who may benefit from site‐specific treatment. Pending results from the randomized CUPISCO trial will also provide more clarity on the role of molecularly directed therapy compared with empirical chemotherapy.^25^ Given that it usually takes several weeks to receive molecular profiling results,^27^, ^31^ the need for an optimal globally employable strategy for timely identification of these patients will remain a critically important issue.

The strengths of our study are inclusion of only patients who underwent the recommended workup, including imaging and diagnostic tissue biopsy confirming CUP. This data adds to the limited real‐world experience of CUP patients seen in a contemporary setting and highlights the lack of improvement in survival outcomes over time. This study provides insight into the role of site‐specific and empirical treatment for CUP patients and reinforces the challenges reported in previous CUP studies including population heterogeneity. Conclusions are limited by the retrospective design with the inherent risk of selection bias and small numbers within subgroup analyses. Our cohort only included patients from metropolitan centers and tissue of origin assignment was skewed towards poor prognosis tumors namely, upper gastrointestinal malignancies, which may not extrapolate to the patient population seen at other centers. We also did not exclude patients based on the absence of mammography, because this is not universally recommended in the workup of CUP patients and, in autopsy studies, breast‐like CUP remains rare.^32^ Further, relying on ICD codes may have missed identification of all CUP patients, especially in instances where clinicians may have assigned a tissue of origin despite patients satisfying the diagnostic criteria for CUP. Finally, given the time period and location in which our study was conducted, we did not have sufficient statistical power to evaluate the impact of early molecular testing and actionable treatment alterations, which has been suggested as a means to improve outcomes and rationalize treatment options, including targeted and immunotherapies for this patient population.^16^, ^33^ Such testing is likely to become increasingly relevant with further advent of molecularly guided and tumor agnostic treatment strategies.

## 5 CONCLUSIONS

In conclusion, in this retrospective study of patients fulfilling the updated ESMO diagnostic criteria for CUP, prognosis remains poor for patients with unfavorable risk. Site‐specific therapy did not result in improved survival outcome in a heterogenous patient population, many of whom had presumed upper gastrointestinal malignancies. Poor performance status and rapid symptomatic decline precluded systemic treatment and were associated with poor survival outcomes. This reinforces the need for timely investigation for this group of patients and an unmet need for further treatment options.

## AUTHOR CONTRIBUTIONS

**Emma L. Boys:** Conceptualization (equal); data curation (lead); formal analysis (lead); project administration (equal); writing – original draft (lead); writing – review and editing (equal). **Bo Gao:** Project administration (supporting); supervision (equal); writing – review and editing (equal). **Peter Grimison:** Project administration (supporting); supervision (equal); writing – review and editing (equal). **Sarah Sutherland:** Supervision (equal); writing – review and editing (equal). **Karen L. MacKenzie:** Formal analysis (supporting); supervision (equal); writing – review and editing (equal). **Roger R. Reddel:** Conceptualization (equal); funding acquisition (lead); project administration (lead); supervision (equal); writing – review and editing (equal). **Jia Liu:** Conceptualization (equal); formal analysis (supporting); supervision (equal); writing – review and editing (lead).

## FUNDING INFORMATION

E.B. is supported by the Royal Australasian Physicians Arnott Scholarship in Cancer Research and the ICPMR Jerry Koutts Scholarship. ProCan^®^ is supported by the Australian Cancer Research Foundation, Cancer Institute New South Wales (NSW) (2017/TPG001, REG171150), NSW Ministry of Health (CMP‐01), The University of Sydney, Cancer Council NSW (IG 18–01), Ian Potter Foundation, the Medical Research Futures Fund (MRFF‐PD), National Health and Medical Research Council (NHMRC) of Australia European Union grant (GNT1170739, a companion grant to support the European Commission's Horizon 2020 Program, H2020‐SC1‐DTH‐2018‐1, “iPC—individualizedPaediatricCure” [ref. 826121]), and National Breast Cancer Foundation (IIRS‐18‐164). Work at ProCan^®^ is done under the auspices of a Memorandum of Understanding between Children's Medical Research Institute and the U.S. National Cancer Institute's International Cancer Proteogenomics Consortium (ICPC), that encourages cooperation among institutions and nations in proteogenomic cancer research in which datasets are made available to the public.

## CONFLICT OF INTEREST STATEMENT

J.L: Research funding: MSD; honorarium: MSD, Specialized Therapeutics; travel reimbursement: Starpharma, ImmVirX. The other authors declare no conflict of interest.

## CONSENT

Patient consent was waived given the retrospective and low‐risk nature of this study.

## Supporting information


Data S1.

## Data Availability

The data that support the findings of this study are available on request from the corresponding author. The data are not publicly available due to privacy or ethical restrictions.
