# Supplementary material for: Retrospective analysis of clinical characteristics and outcomes of patients with carcinoma of unknown primary from three tertiary centers in Australia
Source: Cancer Med. 2024 Mar 25;13(6):e7052. doi: 10.1002/cam4.7052 (PMC10961596; doi:10.1002/cam4.7052)
Supplement: Supplementary file 1 — Data S1. [file CAM4-13-e7052-s001.docx]

**Supplementary Tables:**

| **Favorable CUP Subsets** | **Unfavorable CUP Features** |
| --- | --- |
| Solitary metastatic lesion or oligometastatic disease amenable to local treatment | Other patients who do not fit into defined favorable CUP subsets e.g.  Metastatic adenocarcinoma to the liver or other viscera  Multiple brain metastases  Squamous cell carcinoma of the abdomen or pelvis |
| Women with solitary axillary lymph node metastases |  |
| Women with peritoneal disease from serous papillary adenocarcinoma |  |
| Squamous cell carcinoma of non-supraclavicular cervical lymph nodes |  |
| Men with blastic bone metastases and/ or PSA expression (IHC or serum). |  |
| Adenocarcinoma with colorectal IHC pattern (CK7 negative, CK20 positive, CDX2 positive) |  |
| Carcinoma with histological and/ or IHC profile consistent with renal cell carcinoma |  |

**Supplementary Table 1:** Classification of carcinoma of unknown primary patients. Adapted from the European Society of Medical Oncology 2023 guidelines (1). CK, cytokeratin; CDX2, caudal-type homeobox transcription factor 2; CUP, carcinoma of unknown primary; IHC, immunohistochemistry; PSA, prostate specific antigen.

**Supplementary Figures:**

**Treatment**

**No Systemic Treatment**

**Systemic Treatment**

**Survival Probability**


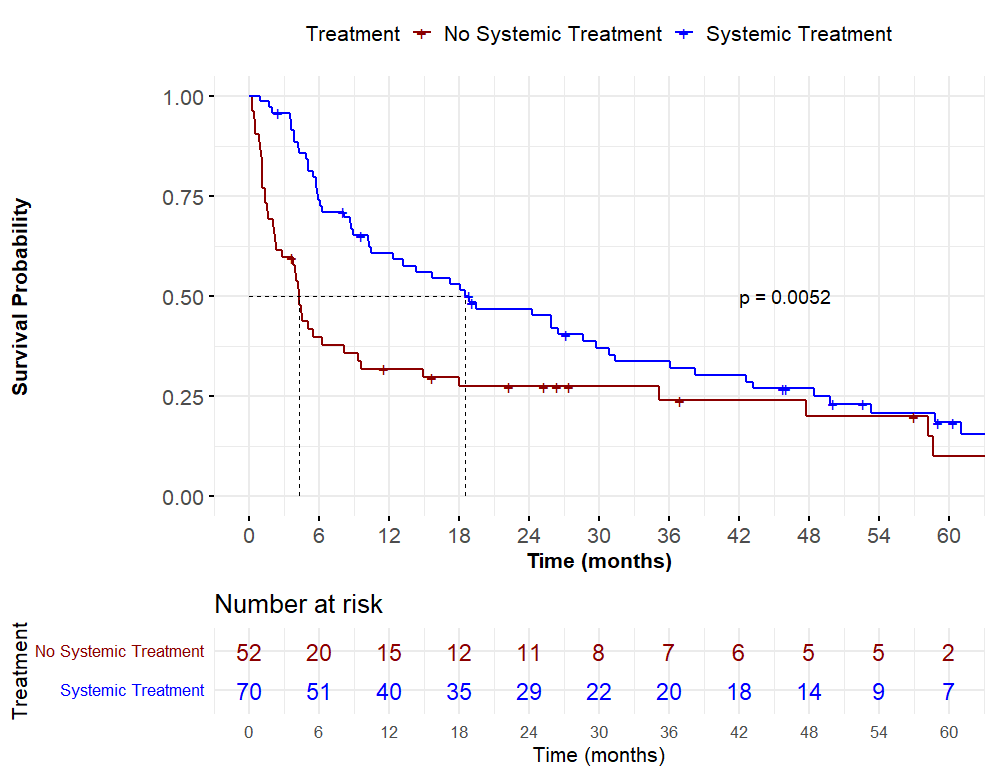

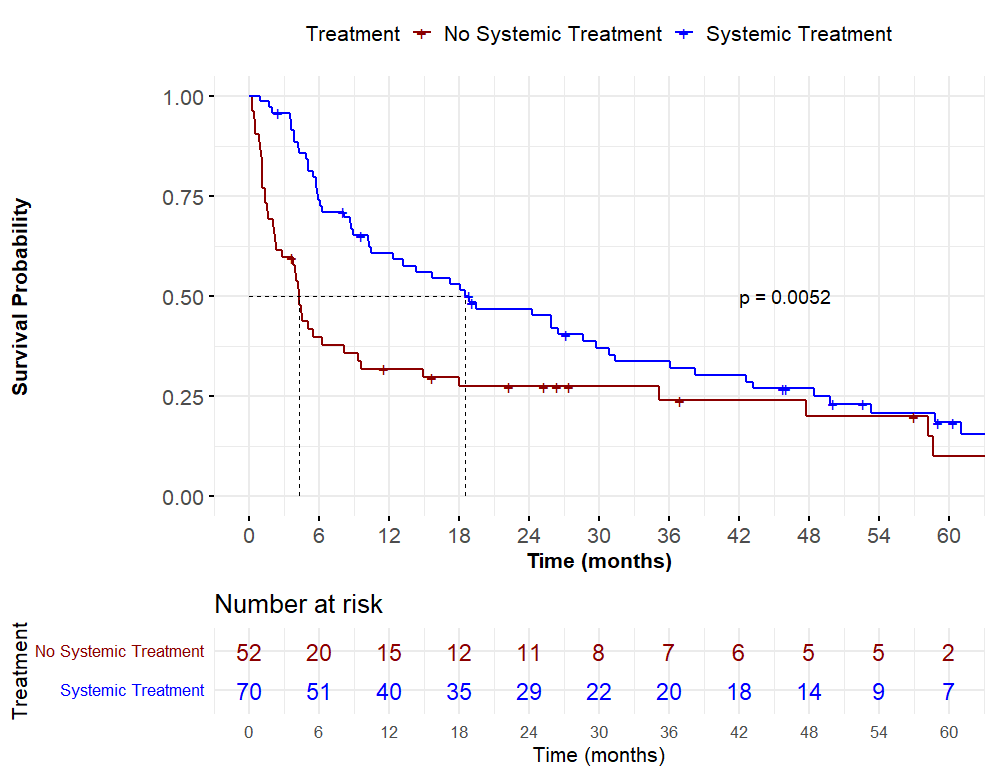


**Supplementary Figure 1:** Overall survival with receipt of systemic therapy (median 18.5, 95% CI 12.3–30.9 months) compared to no systemic therapy (median 4.3, 95% CI 2.3–9.3 months).

**Treatment**

**Chemotherapy**

**Targeted or Immunotherapy**

**Survival Probability**


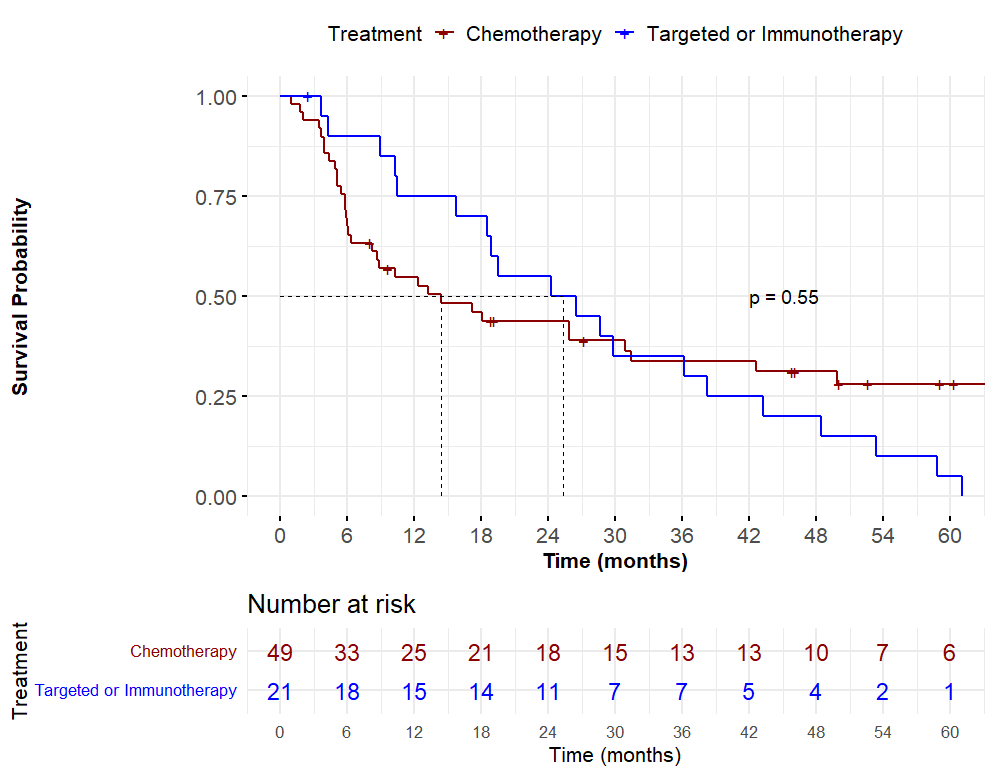

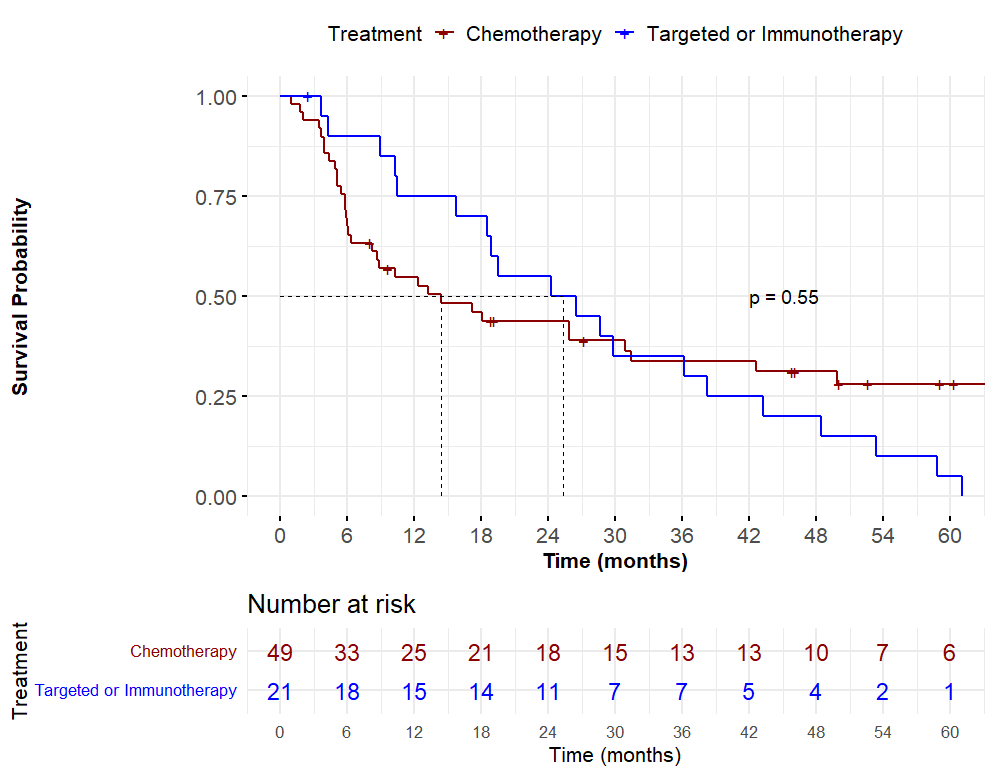

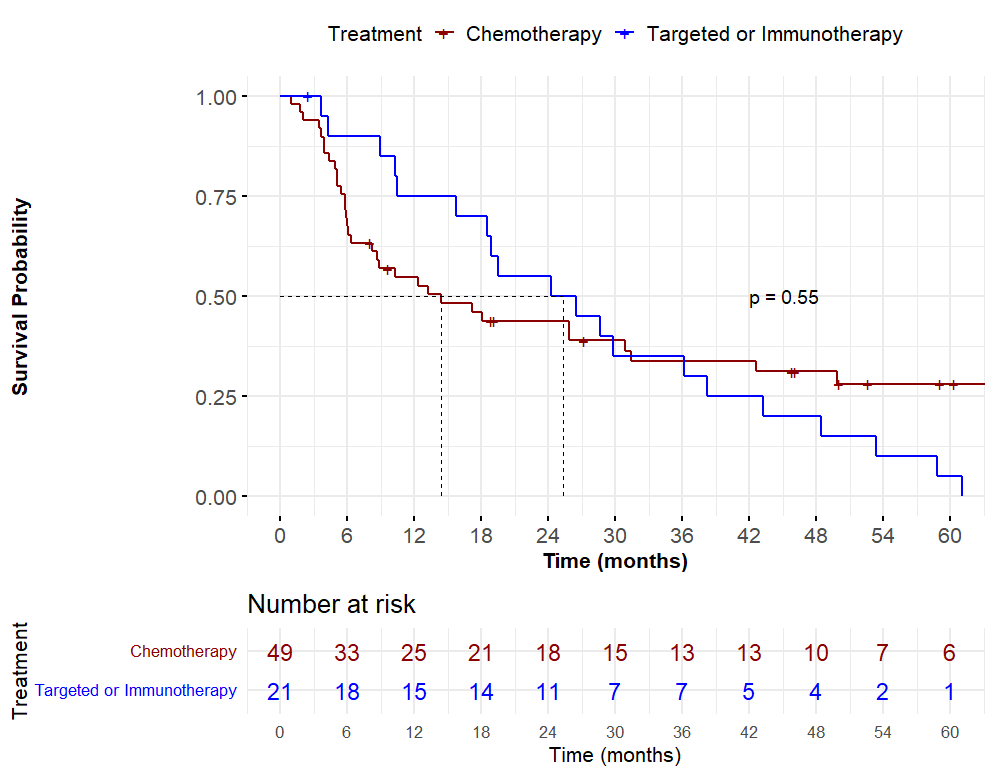


**Supplementary Figure 2:** Overall survival with receipt of targeted or immunotherapy (median 25.3, 95% CI 18.5–43.2 months), compared with chemotherapy alone (median 14.3 months, 95% CI 8.3 – 42.6 months).

**Favorable**

**Unfavorable**

**ESMO Risk Category**

**Survival Probability**


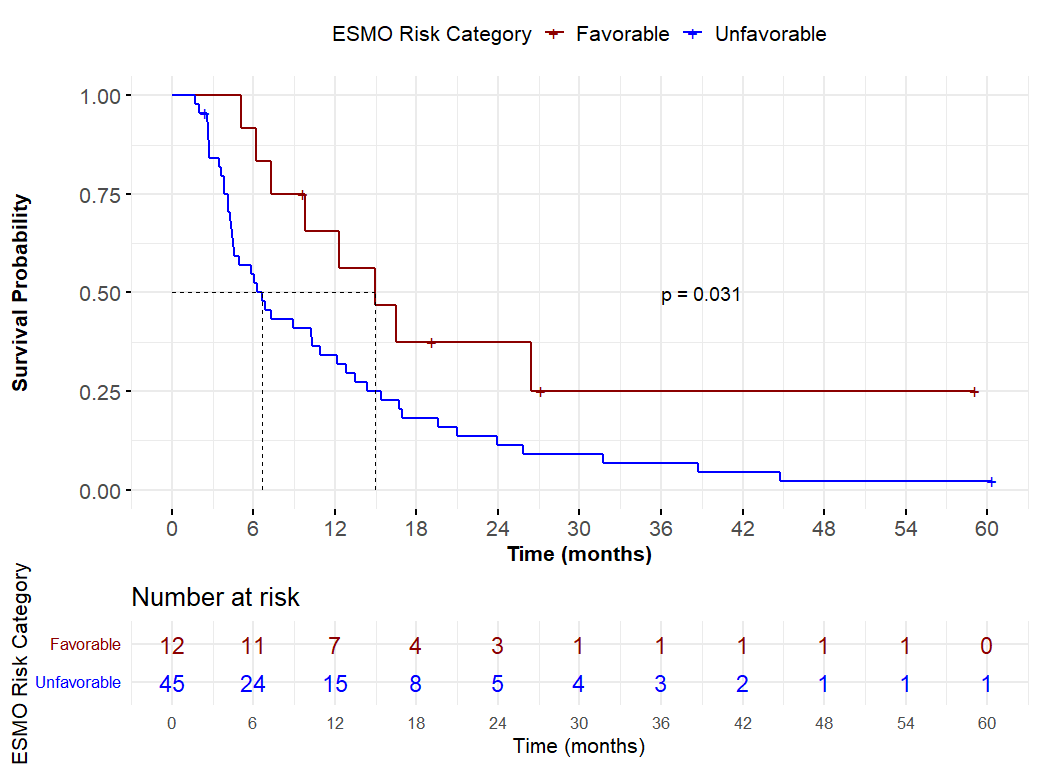

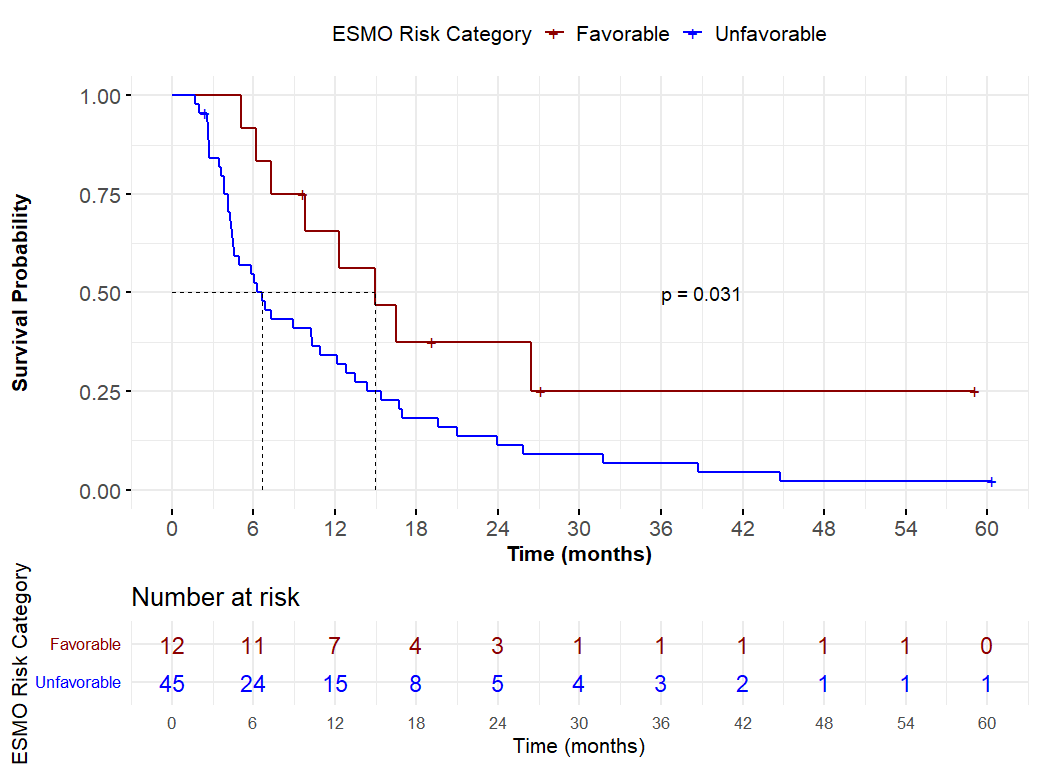

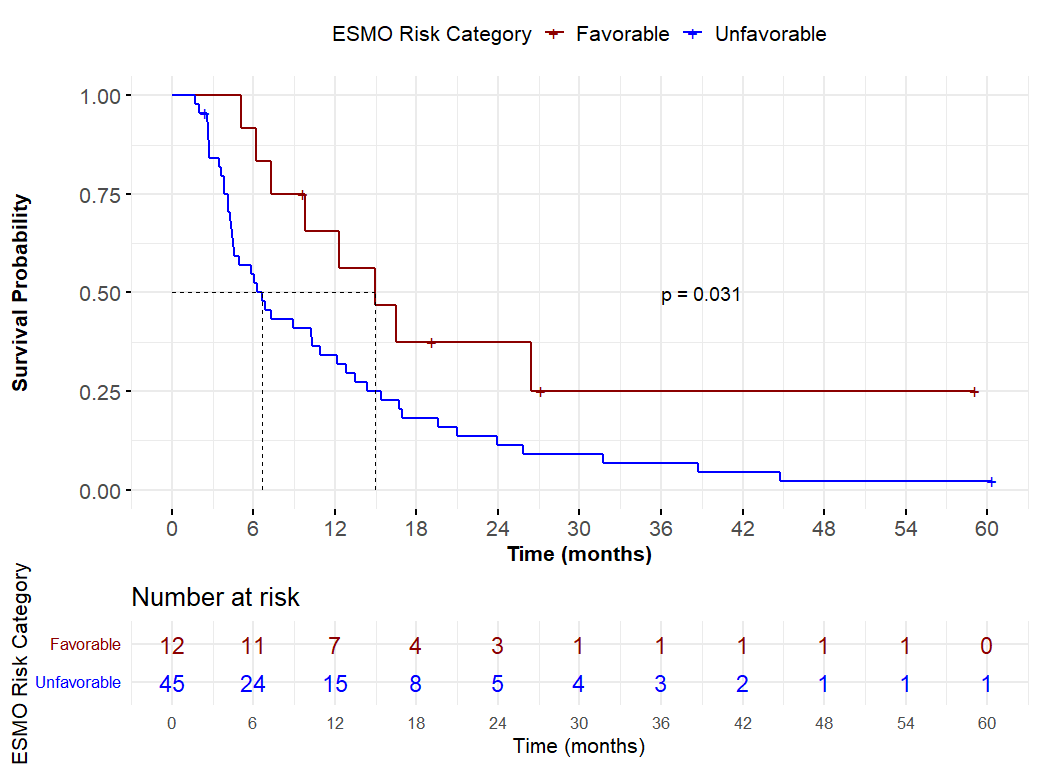


**Supplementary Figure 3:** Progression free survival in patients with non-squamous histology who received systemic treatment stratified by European Society of Medical Oncology risk category. Favorable risk (median 15.0, 95% CI 9.8–no estimate months) was associated with improved progression free survival compared to unfavorable risk patients (median 6.7, 95% CI 4.5-12.1 months).


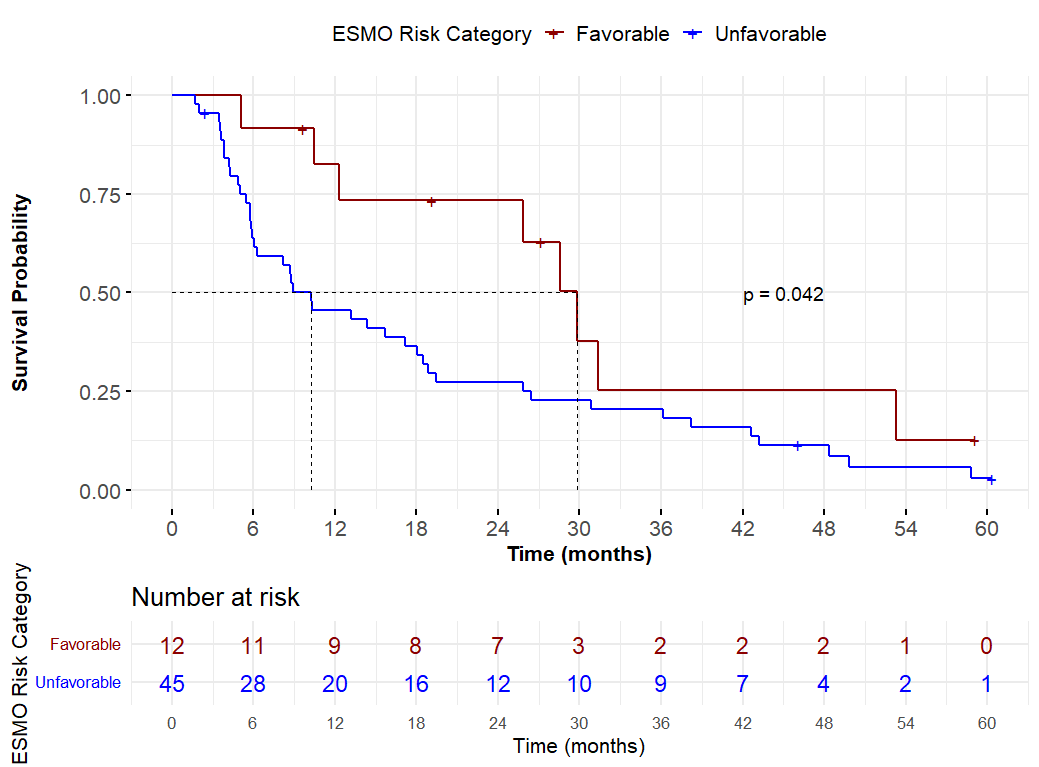

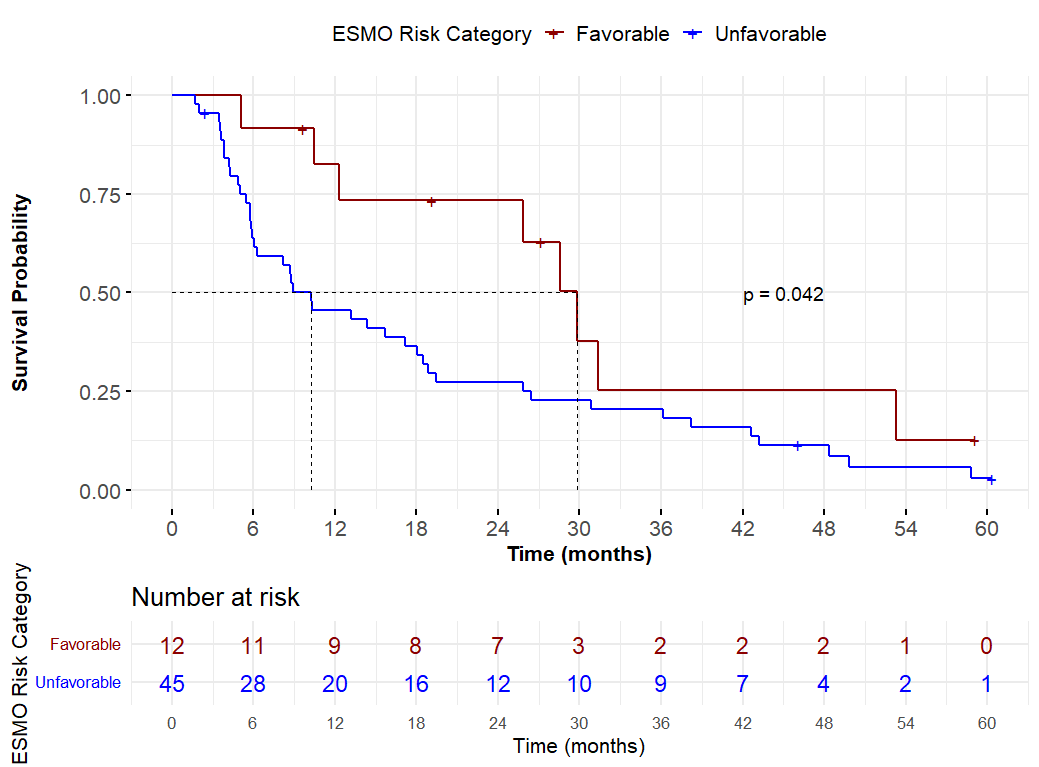


**Favorable**

**Unfavorable**

**ESMO Risk Category**

**Survival Probability**


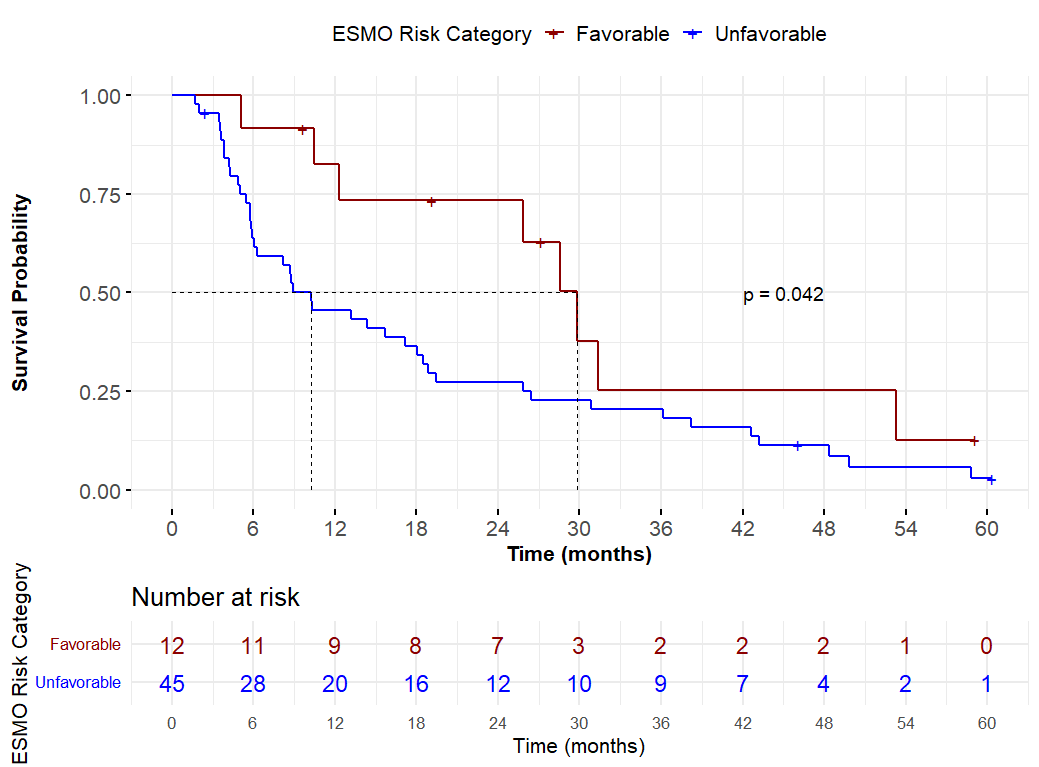


**Supplementary Figure 4:** Overall survival in patients with non-squamous histology who received systemic treatment stratified by European Society of Medical Oncology risk category. Favorable risk was associated with improved overall survival (median 29.8, 95% CI 25.0 –no estimate months) compared to unfavorable risk (median 10.2, 95% CI 6.1–18.5 months).


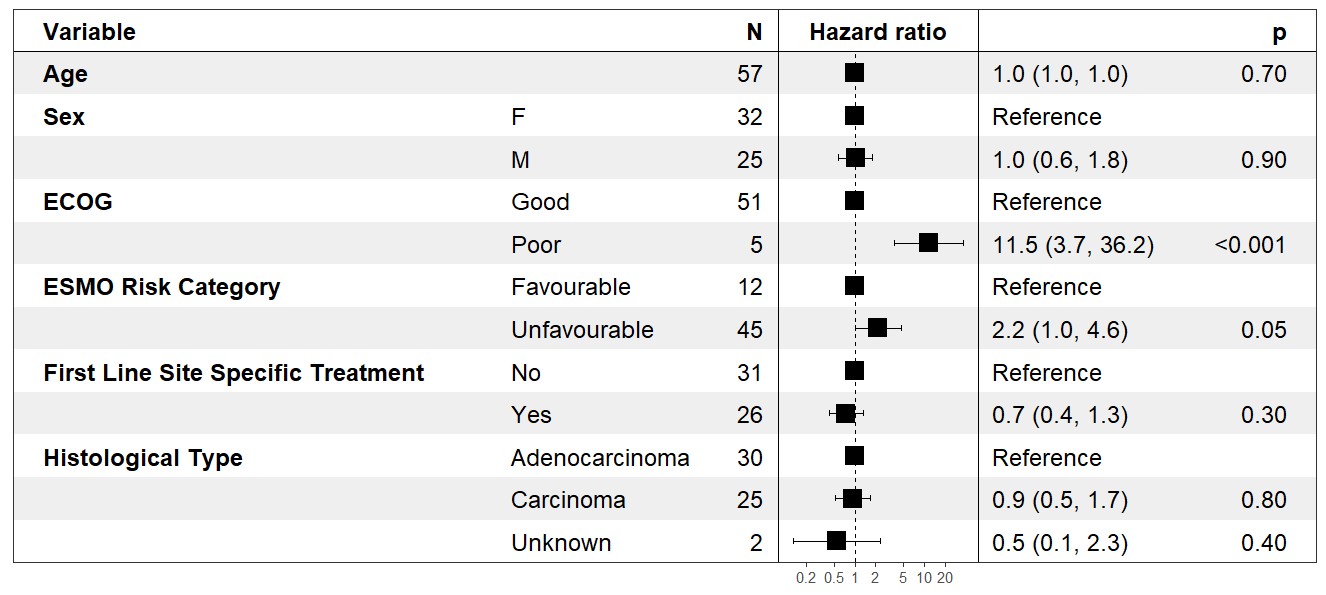
 **Supplementary Figure 5:** Univariable Cox regression proportional hazard analysis of clinical variables associated with OS in patients with non-squamous histology who received systemic treatment.


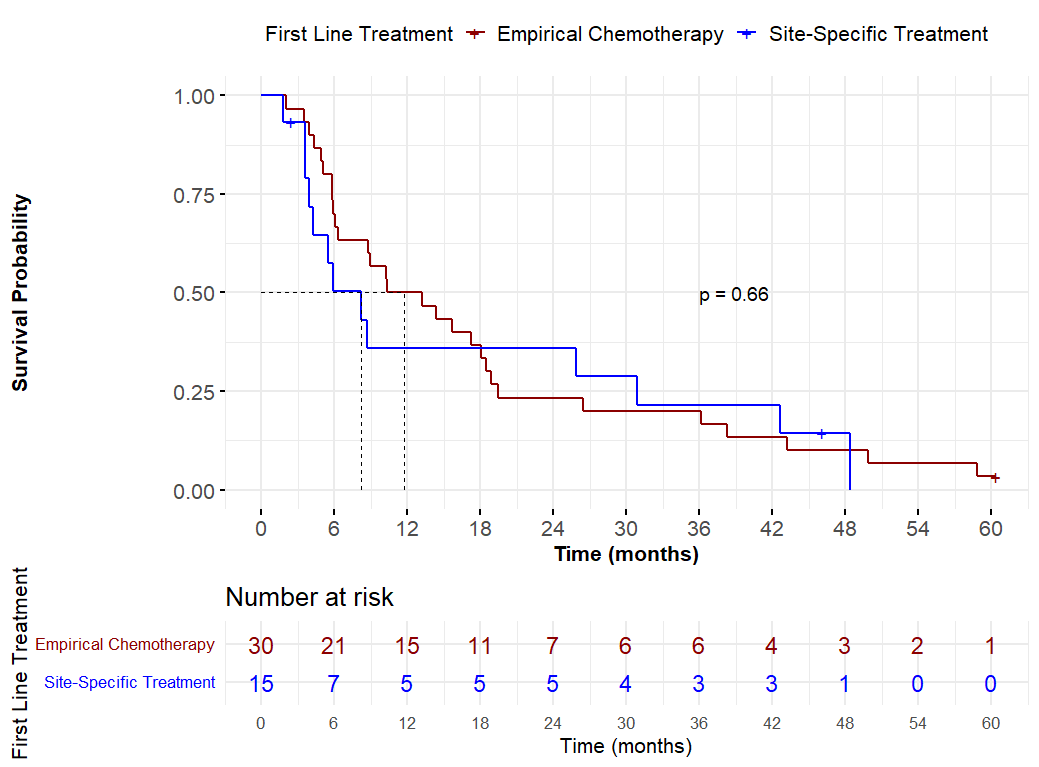


**Empirical**

**Site-Specific**

**First Line Treatment**

**Survival Probability**


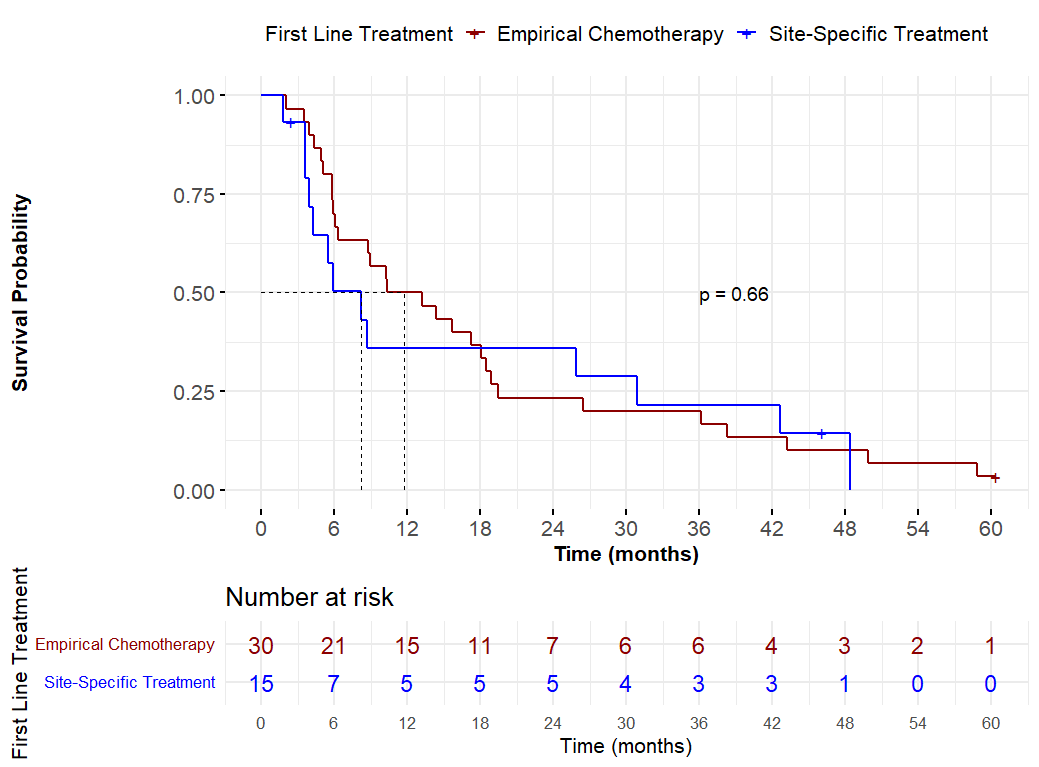

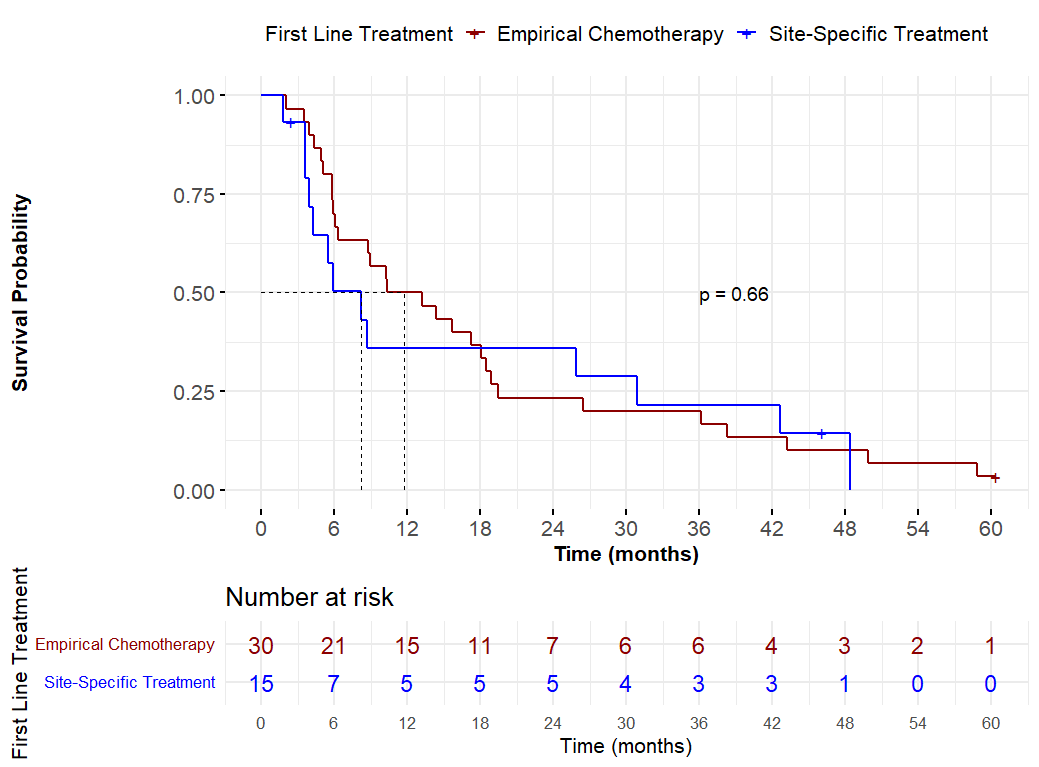


**Supplementary Figure 6:** Overall survival in unfavorable risk patients (non-squamous histology only) stratified by receipt of site specific (median 8.2, 95% CI 4.2–no estimate months) and empirical chemotherapy (median 11.8, 95% CI 6.3–18.9 months).
